# Correction to “Coronavirus Nsp3 Hijacks CLTC to Modulate Autophagosome Nucleation for Promoting DMV Formation and Viral Replication”

**DOI:** 10.1002/advs.76262

**Published:** 2026-06-26

J. Xu, H. Li, P. Liu, et al. “Coronavirus Nsp3 Hijacks CLTC to Modulate Autophagosome Nucleation for Promoting DMV Formation and Viral Replication.” *Advanced Science* 13, no. 24 (2026): e21626, https://doi.org/10.1002/advs.202521626.

**Error Description**:

In paragraph 3 of the “Results” section, the text “Immunoprecipitation analysis revealed that the N‐terminal domain (1–494 aa) of CLTC was necessary for its interaction with nsp3. However, the Distal Leg domain (495–1073 aa) also exhibited detectable binding (Figure 6E). The Proximal Leg & Hub domain showed no interaction. These results indicate that the primary interaction interface resides within the N‐terminal domain, while the Distal Leg may contribute to or stabilize the binding.” was incorrect.

**Correction**:

We hereby correct the description as follows: “Immunoprecipitation analysis revealed that the Proximal Leg & Hub domain (1074–1675 aa) of CLTC co‐precipitates with nsp3, as indicated by a specific ∼70 kDa band in Figure 6E, demonstrating that this domain is involved in the interaction with nsp3.”

**Impact**:

We have carefully re‐evaluated the paper and confirm that this error does not affect the overall conclusions of the study. Moreover, the figure description will not alter the framework, data logic, or core findings of the original manuscript.

We apologize for this error.

